# Factors associated with mammographic breast density among women in Karachi Pakistan

**DOI:** 10.1186/s12905-021-01538-4

**Published:** 2021-12-31

**Authors:** Uzma Shamsi, Shaista Afzal, Azra Shamsi, Iqbal Azam, David Callen

**Affiliations:** 1 School of Medicine, University of Adelaide, Adelaide, Australia; 2 Department of Community Health Sciences, Aga Khan University, Karachi, Pakistan; 3 Department of Radiology, Aga Khan University, Karachi, Pakistan; 4 Department of Gynecology and Obstetrics, Combined Military Hospital, Karachi, Pakistan

**Keywords:** Breast cancer, Mammographic breast density, Age, Benign breast disease, Body mass index

## Abstract

**Background:**

There are no studies done to evaluate the distribution of mammographic breast density and factors associated with it among Pakistani women.

**Methods:**

Participants included 477 women, who had received either diagnostic or screening mammography at two hospitals in Karachi Pakistan. Mammographic breast density was assessed using the Breast Imaging Reporting and Data System. In person interviews were conducted using a detailed questionnaire, to assess risk factors of interest, and venous blood was collected to measure serum vitamin D level at the end of the interview. To determine the association of potential factors with mammographic breast density, multivariable polytomous logistic regression was used.

**Results:**

High-density mammographic breast density (heterogeneously and dense categories) was high and found in 62.4% of women. There was a significant association of both heterogeneously dense and dense breasts with women of a younger age group < 45 years (OR 2.68, 95% CI 1.60–4.49) and (OR 4.83, 95% CI 2.54–9.16) respectively. Women with heterogeneously dense and dense breasts versus fatty and fibroglandular breasts had a higher history of benign breast disease (OR 1.90, 95% CI 1.14–3.17) and (OR 3.61, 95% CI 1.90–6.86) respectively. There was an inverse relationship between breast density and body mass index. Women with dense breasts and heterogeneously dense breasts had lower body mass index (OR 0.94 95% CI 0.90–0.99) and (OR 0.81, 95% CI 0.76–0.87) respectively. There was no association of mammographic breast density with serum vitamin D levels, diet, and breast cancer.

**Conclusions:**

The findings of a positive association of higher mammographic density with younger age and benign breast disease and a negative association between body mass index and breast density are important findings that need to be considered in developing screening guidelines for the Pakistani population.

## Background

Mammography (MMG) is the baseline investigation used for screening and diagnosis of breast cancer, which remains responsible for more than 500,000 deaths each year worldwide [1]. Mammographic breast density (MBD) refers to the relative amount of dense tissue in an entire breast. Dense tissue comprises of connective and epithelial tissue including glandular parenchyma and hinders X-Ray transmission and therefore, appears dense/white on mammography. Fatty parenchyma allows unhindered X-ray transmission and hence appears darker/lucent on a mammogram. Dense breast tissue results in masking for breast cancer and hence the mammographic sensitivity is reduced with increasing MBD [2]. Increased MBD is reported to decrease the sensitivity of screening mammography by 48%, in comparison to the 78% mammographic sensitivity of the entire sample of the study [3]. Moreover, MBD has been recognized as an independent risk factor for breast cancer incidence and recurrence [4]. More than 50% of women in less than 50 years of age in the USA are reported to have dense breasts [5]. Therefore, there has been a lot of interest in the evaluation of factors associated with MBD including the role of environment and genetics and the causal relationship between breast cancer and MBD.

Several factors can affect MBD like age, heredity, parity, ethnicity, diet, hormonal replacement therapy (HRT), and the molecular subtypes of breast cancer [6, 7]. An increasing parity status has been inversely associated with MBD and the percent breast density [8]. MG del Carmen et al. compared breast density among different races and reported MBD to be lowest in white and African American women and highest in Asian women [9]. Race and ethnicity have been explored and identified as important determinants of MBD in another study too [10]. Though race and ethnicity are relevant to breast density, other factors such as diet, and environmental exposures are also important determinants of mammographic density and hence the risk of breast cancer is different in different ethnic groups [11]. There is an inverse association of body weight with the percentage of MBD in both pre and post-menopausal women due to more fat in the breast. It was recently confirmed in another retrospective study showing association of surgical weight loss with a decrease in breast density [12]. Few other studies reported a higher intake of fats, protein, alcohol, and hormonal replacement therapy (HRT) with higher MBD [13, 14]. Tamoxifen, however, reduced MBD in short term, and in a randomized control trial, a 10% reduction in MBD with a 63% breast cancer risk reduction was reported [15]. MBD is also reported to be heritable and has shown a correlation coefficient of 0.74 in monozygotic twins in comparison to 0.38 in dizygotic twins [16]. However, the exact influence of the environment and individual’s behavior on this heritable effect is still not clearly known.

There is no organized (service) screening program in Pakistan and most of the mammography done is opportunistic (individual) screening [17]. Due to limited screening mammography units and screening practices, there is a paucity of epidemiological data on MBD prevalence and factors associated with MBD in Pakistan. Therefore, the joint effects of sociodemographic and other factors associated with MBD among Pakistani women remain unclear. The main objective of this study was to evaluate the factors associated with MBD including serum level of vitamin D, intake of different food categories, physical activity, body mass index BMI, and other risk factors of breast cancer. Another objective was to assess the association of MBD with breast cancer among Pakistani women.

## Materials and methods

The source population of this study is from a multicenter hospital-based case-control study which was conducted at two large tertiary care hospitals of Karachi, the details of the study and the questionnaire used are described in the previously published article [18]. Briefly, 477 women with complete records of MBD were extracted from the main study. We included women of ages 30–74 years subjects from the main study who underwent a screening mammogram between 2015 and 2019. There were 178 breast cancer cases and 299 controls who went diagnostic and screening (annual and biennial) mammography. Women were included even if they had multiple mammograms. Women were excluded if they were unable to complete the interview due to any sickness or been living outside Pakistan for more than a year. We also excluded women if mammographic breast density information was unavailable. All mammograms were taken before breast cancer diagnosis among cases. All subjects completed an interview-based questionnaire that included information on age, education, socioeconomic status, parity, age of mother at first birth, breastfeeding, age at menarche and menopause, age of mother at first birth, history of any comorbid or benign breast disease, family history of breast cancer. Menopausal status was either premenopausal or postmenopausal. Participants also reported the average number of hours per week, engaged in physical activity of different intensities for at least 10 min, like vigorous exercise or moderate exercise of household activities like mopping, etc., and walking. BMI (kg/m2) and tumor characteristics were recorded from medical files and reports. Women with missing information of BI-RADS (Breast Imaging-Reporting and Data System) density were excluded. All consenting participants were interviewed, after informed consent, in a separate room to ensure privacy using a structured questionnaire.

### Mammography measurement details

Two view mammography was performed for all patients comprising of medio-lateral oblique (MLO) and cranio-caudal (CC) views on a computed radiography (CR) system. In the CR system, the X- Rays passing through the breast, grid, and cassette cover are absorbed by the plate reader system that comprises photostimulable storage phosphor (PSP). An electronic latent image is produced on the PSP due to the local absorption of X-ray energy that varies with the anatomical variation of breast parenchyma. The cassette is subsequently placed in the reader that captures the information and converts it to a digital signal that is finally displayed at the workstation [19]. The soft copies of the mammographic images were downloaded and reviewed at picture archiving and communication system (PACS) by breast imagers, and qualitative assessment of mammographic density was done by dedicated radiologists with years of experience in interpreting mammography and breast density.

**Fig. 1:**
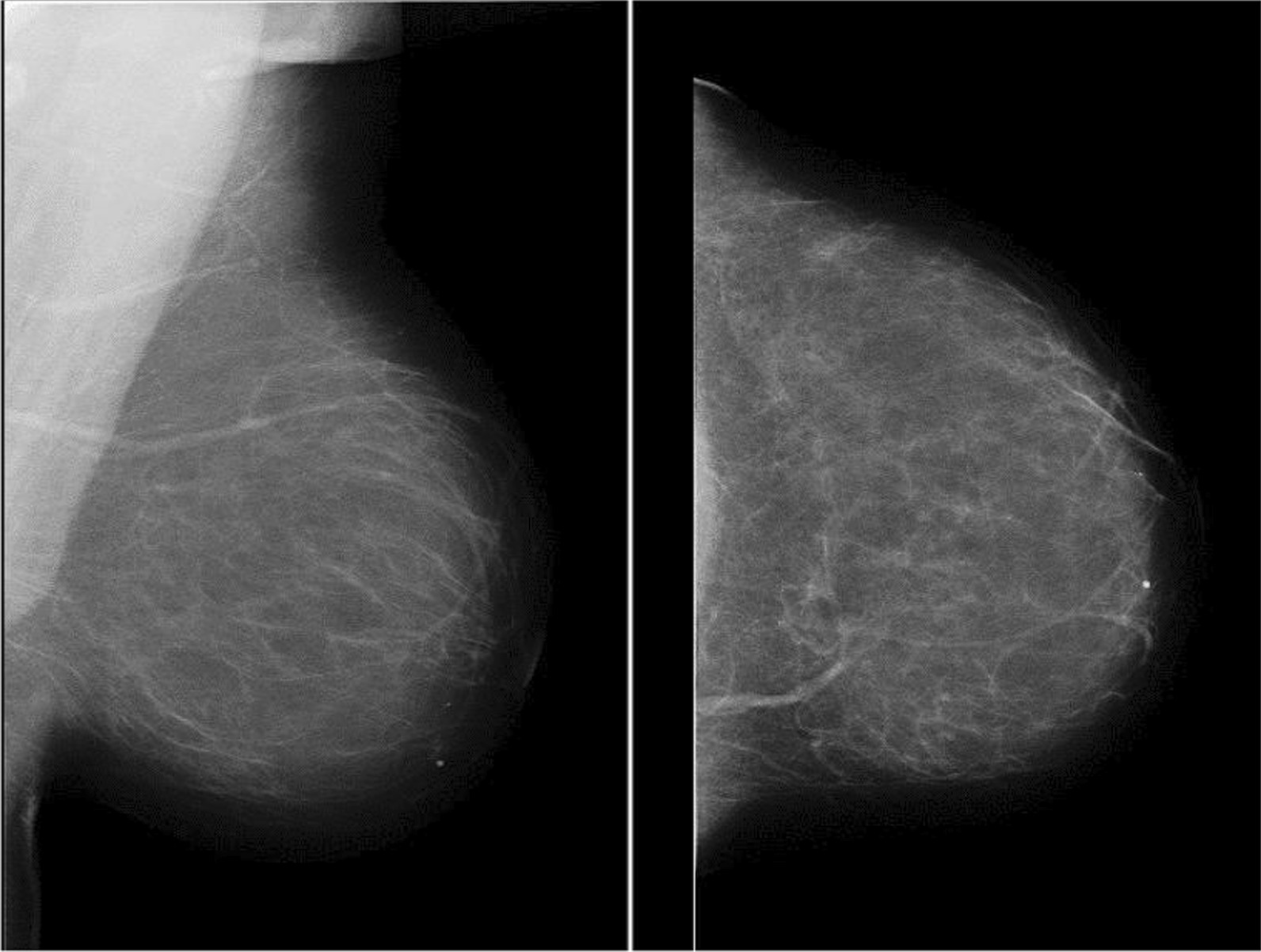
MLO and CC views of left breast showing fatty breast parenchyma

**Fig. 2:**
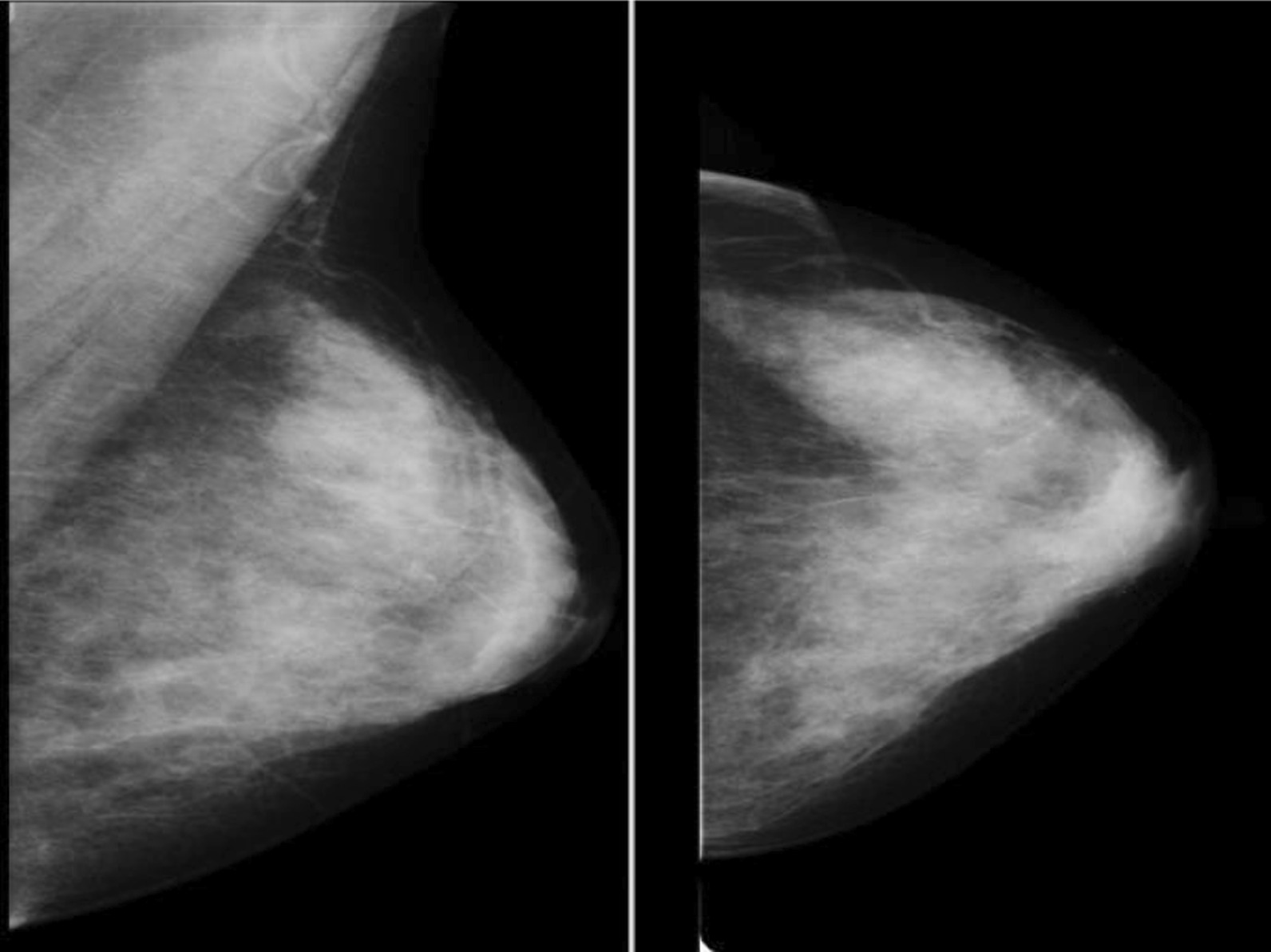
MLO and CC views of left breast showing dense breast parenchyma

### Assessment of mammographic breast density (MBD)

Using the American College of Radiology Breast Imaging Reporting and Data System (ACR BI-RADS 5th edition), the mammographic breast density on mammograms was categorized as; Category 1, Predominantly fatty (less than 25% glandular); category 2 for scattered fibroglandular (25–50% glandular); category 3 for heterogeneously dense (51–75% glandular) and category 4 for dense breast parenchyma (more than 75% glandular) [20]. Categories 3 and 4 both are high MBD (Figs. 1 and 2 showing fatty and dense breast parenchyma). Analyses were restricted to patients with an available BI-RADS measurement. The density measurements of the breast contralateral to the tumor were used to avoid a distortion of measurements due to the tumor itself.

### Dietary intake assessment with a Food Frequency Questionnaire (FFQ)

Dietary intake assessment was done by using the validated food frequency questionnaire FFQ [21]. Intake frequency was categorized into 7 groups and category for each food item was converted to daily intake. Each participant was also asked about their average portion size/common serving size of the food. The intake frequencies were multiplied by standard portion size to calculate servings per day of all food items. All the food items were grouped into six components including fruits, vegetables, dairy, grains, white meat, red meat, and plant proteins and total servings/day was calculated for each food category.

### Measurement of serum 25 (OH)D level

After the interview, blood samples were collected from the study participants and serum 25 (OH)D level was measured using ELISA.

### Tumor characteristics

Histopathology and estrogen receptor (ER), progesterone receptor (PR), and human epidermal growth factor receptor (HER2/neu) status of breast cancer cases were retrieved from medical records [22].

The ethical approval was obtained by the Human Research Ethics Committee of the University of Adelaide and the Ethical Review Committees of two hospitals in Karachi Pakistan: Aga Khan University Hospital AKUH and Karachi Institute of Radiation and Nuclear Medicine Hospital KIRAN. Patients who were literate read and signed the informed consent form and informed consent was obtained verbally from those who could not read or write.

### Statistical analysis

Multinomial logistic regression models were applied to compute odds ratios (ORs) and 95% confidence intervals (CIs) for the MBD categories. The fatty and scattered fibroglandular tissue categories were merged and used as a reference. Multivariate models were adjusted for variables found to be significantly associated with breast density and known risk factors for breast cancer such as age, BMI, age at menarche and menopause in the postmenopausal group, parity, and family history of breast cancer among first-degree relatives, BMI, and the food categories. SPSS 22.0 software (IBM Statistics, Armonk, New York, USA) was used for the analysis.

## Results

**Fig. 3:**
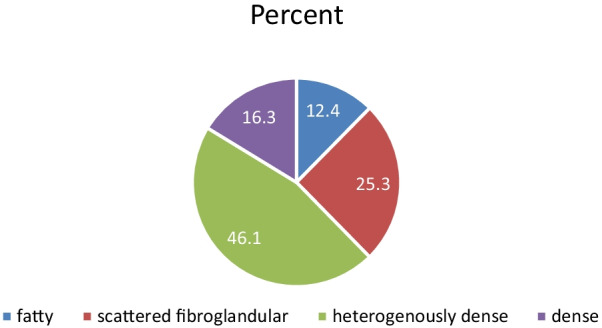
Pie chart depicting the distribution of MBD among Pakistani women

The mean age of the study participants was 46.2 years (SD 11.7). Hundred and eighty (37.3%) women had breast biopsy-proven breast cancer and 297 (62.7%) of the women had normal or benign breast disease. Figure 3 shows that high density MBD (heterogeneously and dense categories) accounted for 62.4% of all participants.

### Characteristics of study participants

Table 1 shows that there were significant differences in age, education, parity, breastfeeding, body weight, BMI, parity, history of breastfeeding, history of comorbid, menopausal status, among women of different breast densities. In both heterogeneously dense and dense groups, there was a higher percentage of women who were of younger age categories, nullipara, lower parity, higher education, and had a family history of breast cancer.

**Table 1:** Characteristics of study participants by mammographic breast density among women in Karachi, Pakistan (n = 477)

Variables	Category	Fatty/fibroglandular (n = 178)	Heterogeneously dense (n = 198)	Dense (n = 101)	p value*
		n (%)	n (%)	n (%)	
Age groups (years)					< 0.001
	< 35	7 (3.9)	12 (6.1)	13 (12.9)	
	35–45	30 (16.9)	69 (34.8)	46 (45.5)	
	46–54	57 (32.0)	51 (25.8)	29 (28.7)	
	55 and above	84 (47.2)	66 (33.3)	13 (12.9)	
Education					0.023
	< Grade8	33 (18.6)	31 (15.7)	13 (12.9)	
	Grades 8–12	65 (36.7)	54 (27.4)	24 (23.8)	
	> Grade12	79 (44.6)	112 (56.9)	64 (63.4)	
Socioeconomic Status SES					0.866
	Upper	43 (25.6)	48 (25.3)	20 (20.0)	
	Middle	114 (67.9)	129 (67.9)	73 (73.0)	
	Lower	11 (6.5)	13 (6.8)	7 (7.0)	
Parity					0.004
	Nullipara	21 (12.5)	18 (9.5)	21 (21.0)	
	1–3	78 (46.4)	112 (58.9)	55 (55.0)	
	> 3	69 (41.1)	60 (31.6)	24 (24.0)	
Age of mother at first live birth (AFB)					0.060
	< 20 years	39 (25.8)	29 (16.2)	11 (13.9)	
	20–29 years	98 (64.9)	126 (70.4)	54 (68.4)	
	> 30 years	14 (9.3)	24 (13.4)	14 (17.7)	
Breastfeeding duration					0.910
	< 12 months	26 (19.3)	35 (21.2)	15 (19.7)	
	> 12 months	109 (80.7)	130 (78.8)	61 (80.3)	
Age at menarche (years)					0.487
	< 12 years	26 (16.3)	22 (12.2)	12 (12.6)	
	12–13 years	95 (59.4)	105 (58.0)	51 (53.7)	
	> 14 years	39 (24.4)	54 (29.8)	32 (33.7)	
History of any comorbid					0.01
	Yes	102 (57.3)	95 (48.0)	39 (38.6)	
	No	76 (42.7)	103 (52.0)	62 (61.4)	
History of benign breast disease					0.001
	Yes	33 (19.9)	61 (32.6)	46 (46.5)	
	No	133 (80.1)	126 (67.4)	53 (53.5)	
Family history of breast cancer					< 0–001
	Yes	48 (28.6)	77 (40.7)	32 (32.0)	
	No	120 (71.4)	112 (59.3)	68 (68.0)	
Menopausal status					< 0.001
	Menopause	125 (74.4)	102 (54.3)	40 (40.4)	
	Premenopause	43 (25.6)	86 (45.7)	59 (59.6)	
Serum vitamin D level (ng/ml)					0.761
	< 20	78 (57.4)	82 (54.7)	47 (54.7)	
	20–30	21 (15.4)	30 (20.0)	19 (22.1)	
	> 30	37 (27.2)	38 (25.3)	20 (23.3)	
Mean BMI (SD)	Menopausal women	30.4 (4.8)	28.4 (4.9)	25.4 (4.2)	< 0.001
	Premenopausal women	27.9 (5.0)	28.2 (4.7)	25.0 (3.8))	< 0.001

BI-RADS: Breast Imaging Reporting and Data System (fatty and scattered glandular dense = categories A and B, heterogeneously dense = C, dense = D); chi-square test*

### Tumor characteristics

There was no significant difference observed in the tumor characteristics among different MBD categories (Table 2).

**Table 2:** Tumor characteristics of breast cancer cases according to mammographic breast density among women in Karachi Pakistan (n = 178)

Hormonal receptor (IHC)	Fatty/fibroglandular (n = 178)	Heterogeneously dense (n = 198)	Dense (n = 101)	p value*
	n (%)	n (%)	n (%)	
*ER*				0.428
Positive	41 (65.1)	59 (73.8)	22 (75.9)	
Negative	22 (34.9)	21 (26.3)	7 (24.1)	
*PR*				0.164
Positive	37 (58.7)	59 (73.8)	19 (65.5)	
Negative	26 (41.3)	21 (26.3)	10 (34.5)	
*Her2*				0.65
Positive	10 (17.5)	16 (23.2)	7 (25.0)	
Negative	47 (82.5)	53 (76.8)	21 (75.0)	
*TNBC*				0.113
TNBC	17 (27)	11 (14.1)	4 (13.8)	
Non-TNBC	46 (73)	67 (85.9)	25 (86.2)	

BI-RADS: Breast Imaging Reporting and Data System (fatty and scattered glandular dense = categories A and B, heterogeneously dense = C, dense = D); TN: triple-negative; ER: estrogen receptor; PR: progesterone receptor; HER2: human epidermal growth factor 2; chi-square test*

There was a positive association of MBD with young age, nulliparity and low parity, family history of breast cancer and history of benign breast disease BBD. Significant protective association of MBD was observed for women of younger age, menopausal status, younger age at first birth (<20 years AFB) and BMI (Table 3).

**Table 3:** Association of factors with mammographic breast density using odds ratios (OR) and 95% confidence intervals (CI), among women in Karachi, Pakistan

Variable	Category	Heterogeneously dense		Dense	
		OR	95%CI	OR	95%CI
Age (years)	< 35	2.18	0.81, 5.85	12.00	4.04, 35.65
	35–45	2.93	1.65, 4.92	9.91	4.71, 20.84
	46–54	1.14	0.69, 1.87	3.29	1.58, 6.86
	55 and above	Ref		Ref	
Socioeconomic status	Upper	0.94	0.38, 2.32	0.73	0.24, 2.17
	Middle	0.95	0.41, 2.22	1.00	0.37, 2.71
	Lower	Ref		Ref	
Parity	Nullipara	0.95	0.47, 1.93	2.79	1.32, 5.91
	< 3	1.63	1.05, 2.53	1.94	1.10, 3.41
	> 3	Ref		Ref	
AFB* (years)	< 20	0.42	0.18, 0.97	0.28	0.10, 0.76
	20–29	0.79	0.38, 1.65	0.54	0.23, 1.22
	> 30	Ref		Ref	
Breastfeeding duration	< 12 months	1.13	0.65, 1.95	1.09	0.55, 2.16
	> 12 months	Ref		Ref	
Age at menarche	< 12yrs	0.61	0.30, 1.23	0.56	0.25, 1.29
	12-13yrs	0.8	0.49, 1.31	0.65	0.37, 1.17
	> 14yrs	Ref		Ref	
Menopause	Menopause	0.38	0.24, 0.59	0.21	0.13, 0.36
	Premenopause	Ref		Ref	
Age at menopause	< 45	0.92	0.48, 1.78	2.02	0.82, 5.01
	> 45	Ref		Ref	
History of benign	Yes	1.72	1.08, 2.73	2.99	1.76, 5.09
	No	Ref		Ref	
Breast cancer	Yes	1.71	0.77, 1.77	0.66	0.39, 1.13
	No	Ref		Ref	
BMI (kg/m^2^)*		0.94	0.90, 0.98	0.80	0.75, 0.86
Physical activity* (h/week)	Walking	0.99	0.89, 1.09	0.95	0.83, 1.09
	Moderate exercise	1.02	0.98, 1.05	1.00	0.96, 1.05
	Vigorous exercise	Ref		Ref	
Vitamin D (ng/ml)	< 20	1.02	0.59, 1.77	1.12	0.58, 2.14
	20–30	1.39	0.68, 2.85	1.67	0.73, 3.82
	> 30	1 (Ref)		1 (Ref)	

Fatty and scattered glandular combined as the reference group. BI-RADS: Breast Imaging Reporting and Data System (fatty and scattered glandular dense = categories A and B, heterogeneously dense = C, dense = D); BMI: body mass index OR: odds ratio; 95% CI: 95% confidence interval AFB Age at first birth* mean (SD)*

### Diet and breast density

There was no association reported between different MBD and food categories of grains, fruits, vegetables, plant proteins, dairy products, white and red meat as shown in Table 4.

**Table 4:** Association between average servings per day of different food categories and mammographic breast density among women in Karachi, Pakistan

Food categories	Heterogeneously dense			Dense		
	OR	95% CI		OR	95% CI	
Grains per day	1.00	0.87	1.16	1.08	0.92	1.27
Vegetables per day	0.98	0.91	1.06	1.04	0.95	1.13
Fruits per day	0.97	0.87	1.09	0.99	0.87	1.13
Dairy products per day	0.84	0.72	0.97	0.93	0.78	1.1
Red meat per day	1.02	0.78	1.33	1.14	0.85	1.53
White meat per day	1.15	0.81	1.62	1.17	0.79	1.75
Plant proteins per day	0.91	0.75	1.11	0.85	0.67	1.07

Fatty and scattered glandular combined as the reference group

OR, odds ratio; 95% CI, 95% confidence interval

Multivariable analysis using multinomial logistic regression (Table 5) shows that women with dense and heterogeneously dense breasts versus fatty and fibroglandular breasts were of a younger age group and had higher benign breast disease BBD. An inverse relationship of BMI with MBD was also observed. There was no association of serum vitamin D levels or breast cancer with breast density.

**Table 5:** Adjusted Odds Ratios (95% CI) for factors associated with mammographic breast density among women in Karachi, Pakistan

Category	Heterogeneously dense MBD		Dense MBD	
Variables	AOR	95% CI	AOR	95% CI
*Age (years)*				
< 45	2.68	1.60, 4.49	4.83	2.54, 9.16
> 45	Ref		Ref	
*History of benign breast disease*				
Yes	1.90	1.14, 3.17	3.61	1.90, 6.86
No	Ref		Ref	
BMI*	0.94	0.90, 0.99	0.81	0.76, 0.87

Ref is fatty/fibroglandular category. BI-RADS: Breast Imaging Reporting and Data System (fatty and scattered glandular dense = categories A and B, heterogeneously dense = C, dense = D)

AOR, adjusted odds ratio; 95% CI, 95% confidence interval; *mean (SD)

AORs adjusted for socioeconomic status, vitamin D, parity, diet, menopausal status/age at menopause, age at first birth, family history of breast cancer, exercise, and case–control status of women

## Discussion

The study findings of significant association of younger age and BBD with higher MBD and protective association of higher BMI are consistent with other studies. Younger age as an important determinant of a high MBD is similar to other studies done in different populations [23–27]. In another study, there was a decline in density reported with age in women with and without breast cancer [28]. It supports the hypothesis that breast density declines with age as younger women have a higher proportion of dense breast tissue compared to older women [6]. In a study done on data from the San Francisco Mammography Registry, breast density decreased with age on an annual basis over the perimenopausal stage [29]. Our findings provide important data on the association of benign breast disease with higher mammographic breast density and are similar to other studies [30–32].

In our study, the inverse relationship between MBD and higher BMI remained the same in both menopausal and premenopausal women. The inverse relationship between BMI and MBD has been reported in previous studies among both menopausal and premenopausal women [23, 33, 34]. In the Minnesota Breast Cancer Family Study, higher BMI was associated with lower breast density among postmenopausal women only [35]. BMI was also inversely related to MBD in Chinese women [36]. An inverse relationship of greater body weight with percentage mammographic density was also reported in premenopausal women, with mammogram showing a larger area of fatty tissue caused by an increased quantity of fat in the breast [37].

Similar to our study findings, a study among Japanese women reported that younger age and BMI were inversely related to high MBD [38]. In another breast cancer family study of 426 families, younger age and lower BMI were associated with increased MBD in both premenopausal and postmenopausal women. Hormone replacement therapy among postmenopausal women was also associated with MBD but this association of MBD with hormone replacement therapy was not seen in the current study as HRT use is very small among Pakistani women. In a study in Norway, volumetric mammographic density (VMD) was inversely associated with age and BMI which is consistent to our study findings [31, 39]. A negative association of high MBD with age, low parity and BMI, was similarly reported by other studies [40, 41].

However, this study observed no association between MBD and breast cancer, and this lack of association has also been reported in a study done in the USA [42] and one in China [42]. A nested case-control study in Ontario, Canada also reported no association of MBD with breast cancer among women with BRCA mutation[43]. Another study at the Massachusetts General Hospital showed that while Asians had the highest breast density, the incidence of breast cancer among them was lesser than that among white women [13]. An observational study of the Mayo Clinic Benign Breast Disease (BBD) cohort failed to find any evidence of an association between MBD and breast cancer risk in women diagnosed with BBD of atypical hyperplasia (AH) type [44]. Similarly, another study done at Johns Hopkins showed that breast cancer was not associated with MRI breast density [45]. The study findings did not show any association of MBD with vitamin D levels, similar to the Nurses’ Health Study [46]. There was also no association observed between MBD and diet.

The high percentage of high MBD (62.4%) in Pakistani women is important information and will be helpful in planning or advising for an organized screening mammography program in Pakistan in future. This is higher than the reported percentage among women in the USA [47] and lower than the reported percentage among women in Jordon [48]. High density breast, on one hand, increases the rates of false-negative diagnosis and on the other hand, there is a requirement of additional tests. The need for additional tests like ultrasound, and MRI increases the sensitivity of screening programs [49] but the cost then becomes too high for the majority of the asymptomatic women to opt for regular screening as there is no health insurance and high poverty in Pakistan.

Significant association of younger age with high MBD in our study is an important finding because ACR guidelines are followed here in the absence of screening guidelines established for Pakistani women where mean age at the time of breast cancer diagnosis reported is also much younger compared to the women in America (46.1 years ± SD 10.1) [50]. Mammographic breast density was also found to be positively associated with BBD and negatively associated with BMI. In univariate analysis, lower parity, breastfeeding, and family history of breast cancer also showed a significant protective association with dense breast. However, in a multivariate analysis performed to adjust for confounding factors, only younger age, BBD, and BMI remained as significant factors associated with high breast density.

### Limitations

Study limitations include small sample size and limited generalizability of the study population due to lack of any organized screening mammography program in Pakistan. Moreover, there was no availability of digital radiography (DR) which was installed in 2019, after the completion of the data collection period of the study. We could not calculate percentage density and absolute dense area due to the lack of availability of the software. There is still a lack of standardization in MBD assessment with high density definitions varying widely from 25 to 75% of dense tissues on mammograms in different studies. Though we tried our best to evaluate MBD with standard mammographic procedures, breast density classification as well as a standardized definition of MBD. Still, some misclassification of MBD could have affected our results since we used visual classification using BI RADS by two experienced radiologists. However, a study reported very good agreement between automatic assessment software of breast density based on artificial intelligence (AI) and visual assessment by a senior and a junior radiologist [51]. The strengths of the study are a good quality of data collection by medical doctors, and comprehensive assessment of all factors associated with MBD.

### Conclusion and recommendations

To our knowledge, this study is the first to report unique distribution of MBD and identify factors associated with MBD in Pakistani women. The findings of positive association of higher mammographic density younger age and BBD and negative association between BMI and breast density are consistent with predictors of mammographic density observed in other populations; however, certain risk factors were not significantly associated with BMD. These are important findings which may be helpful to develop screening guidelines for Pakistani population. Given the current role of breast density in determining breast cancer screening protocols, public health policy, and future research directions, it is important to validate our findings in a larger scale investigation with the advanced technology of the DR system and assess if it is better than screen-film mammography in women with dense breasts.

## Data Availability

Only the data SPSS file has been submitted and mentioned as per requirement.

## Abbreviations

ACR: American College of Radiology
AH: Atypical hyperplasia
AFB: Age at first birth
BI-RADS: Breast Imaging-Reporting and Data System
BMI: Body mass index
BBD: Benign breast disease
CC: Craniocaudal
CR: Computed radiography
DR: Digital radiography
ER: Estrogen receptor
FFQ: Food Frequency questionnaire
HER2: human epidermal growth factor 2
MBD: Mammographic breast density
MLO: mediolateral oblique
PR: Progesterone receptor
SES: Socioeconomic status
